# Is inertial training more effective than traditional resistance training in young healthy males?

**DOI:** 10.3389/fphys.2024.1487624

**Published:** 2024-11-25

**Authors:** Alicja Naczk, Katarzyna Kisiel-Sajewicz, Ewa Gajewska, Piotr Gramza, Tomasz Jędzrzejczak, Mariusz Naczk

**Affiliations:** ^1^ Department of Physical Education and Sport, Faculty in Gorzow Wielkopolski, Poznan University of Physical Education, Poznan, Poland; ^2^ Department of Kinesiology, Wroclaw University of Health and Sport Sciences, Wroclaw, Poland; ^3^ Department of Developmental Neurology, Poznan University of Medical Sciences, Poznan, Poland; ^4^ Department of Innovation, Association of Lubusz Innovation Network, Gorzow Wielkopolski, Poland; ^5^ Department of Applied and Clinical Physiology, Collegium Medicum, University of Zielona Gora, Zielona Gora, Poland

**Keywords:** inertial, resistance, trainings comparison, muscle strength, elbow flexors, knee extensors

## Abstract

**Objectives:**

Inertial training, also called flywheel training is more and more popular among sportsmen. The available data concerning the effectiveness of inertial training compared to conventional resistance strength training are contradictory. The aim of this study was to compare the impact of inertial training (IT) vs. traditional gravity-dependent resistance training (TRT) on elbow flexor and knee extensor strength.

**Methods:**

Twenty-six young, recreationally active males were randomized into IT group (n = 13) or TRT group (n = 13). Both groups performed strength training three times a week for 6 weeks. Before and after training, the maximum force of the trained muscles was evaluated under training conditions (one repetition maximum under gravity-dependent conditions and maximal force under inertial conditions) and isometric conditions. Countermovement jump, squat jump, pull-up test, and limb circumference were also evaluated.

**Results:**

Elbow flexor muscle strength and arm circumference increased significantly in both IT and TRT over the course of training. There were no significant differences in relative muscle strength increases between groups. Knee extensor muscle strength also improved significantly in IT, regardless of the tested conditions, while TRT showed significant changes in one repetition maximum and isometric force but no significant changes in force obtained under inertial conditions. Thigh circumference increased in IT (P ≤ 0.05) but was unchanged in TRT. Jumping abilities improved significantly in both groups, without any differences between groups.

**Conclusion:**

We cannot confirm the superiority of inertial training over traditional resistance training definitively. Nevertheless, inertial training had a slight advantage over traditional resistance training when knee extensor muscle training was considered.

## 1 Introduction

Inertial training is a strength training method that is performed using a specialized gravity-independent device. Studies on the effectiveness of inertial training in young, untrained healthy participants have demonstrated that it is a safe and highly effective training method (Seynnes et al., 2007; Naczk et al., 2016a). Moreover, there are progressively more studies showing that inertial training is an effective strength training method for well-trained professional athletes (Askling et al., 2003; Maroto-Izquierdo et al., 2017a; Naczk et al., 2017). Moreover, inertial exercises are often used for effective rehabilitation in different diseases (Fernandez-Gonzalo et al., 2016; Harris-Love et al., 2021; Naczk et al., 2022) or to improve the quality of life of people with deteriorating locomotor system efficiency (e.g., the elderly) (Brzenczek-Owczarzak et al., 2013; Kowalchuk and Butcher, 2019; Naczk et al., 2020). During inertial training, great muscle tension is maintained during both concentric and eccentric contractions. During traditional resistance training EMG amplitude is markedly lower during eccentric than concentric actions given the same force or load is employed. However, muscle fiber activation during the eccentric action performed with inertial exercise is greater than noted during traditional resistance training (Norrbrand et al., 2008).

Moreover, muscle activity (assessed by EMG amplitude) in inertial exercises is even greater during eccentric contraction than during concentric contraction (Norrbrand et al., 2008). Due to the significant muscle strength increases that develop over relatively short periods of time, the effectiveness of inertial training can be greater than that of traditional resistance training. There is considerable discussion about the superiority of inertial training (also called flywheel training) over traditional resistance strength training. In a summary of their review article, Maroto-Izquierdo et al. (2017b) stated that inertial training appeared to be more effective than traditional resistance exercise in promoting increases in capacities strongly associated with athletic performance. Shortly after publication of that paper, Vicens-Bordas et al. (2018a) in a letter to the editor responded that they believed the methodological shortcomings in the Maroto-Izquierdo et al. (2017b) called its conclusion into question—that inertial flywheel resistance training is superior to traditional weight stack exercises for promoting skeletal muscle adaptations in terms of strength, power, and size in healthy participants and athletes. Maroto-Izquierdo et al. (2018) responded in that letter to the editor that their methodology was satisfactory and their conclusions were justified. Subsequently, Vicens-Bordas et al. (2018b) of this issue; they stated in their conclusions that inertial flywheel resistance training was not superior to gravity-dependent resistance training in improving muscle strength. However, since all cited authors drew their conclusions based on reviews, there is no objective data comparing the effectiveness of inertial training vs. traditional weight training. Some authors have tried to compare the effectiveness of inertial training vs. traditional weight training, but the movement techniques in the two training groups were different and/or the estimations of training loads were not homogeneous (de Hoyo et al., 2015; Greenwood et al., 2007; Onambélé et al., 2008). We assert that the lack of data from a precise, objective, and reliable comparison of the effectiveness of inertial training vs. conventional strength training is probably due to methodological limitations. In gravity-dependent strength training, the training load is determined by the relative value of 1RM, whereas it is not possible to determine the value of 1RM in inertial training. The level of maximum force achieved during inertial exercise depends on both the load used and the speed of movement. Our experiences with inertial training indicate that depending on the individual characteristics of the participant, maximum force is obtained at different values of the applied load and speed of movement. We surmise that this problem has prevented researchers from making a direct, objective comparison between the effectiveness of inertial training and gravity-dependent strength training. To make such a comparison, a novel methodology needs to be developed to determine the load in inertial training, which would be very similar to the load used in traditional weight training. Naczk et al. (2016b) proposed an interesting idea for determining the load in inertial training. They claimed that it is appropriate to use a different load for each participant to reach during inertial exercises but the same maximum speed of movement by all participants. In our opinion, this idea can be modified to create a comparable methodology for studying the differences between inertial training and conventional strength training.

The data concerning the effectiveness of inertial training vs. conventional strength training are contradictory. The aim of this study was to directly compare the effectiveness of inertial training and conventional strength training in young healthy males. It can be assumed that both trainings will be effective in increasing muscle strength and power tested in various conditions. It is possible that due to its specificity, inertial training may be more effective than traditional strength training.

## 2 Materials and methods

### 2.1 Study participants

**FIGURE 1:**
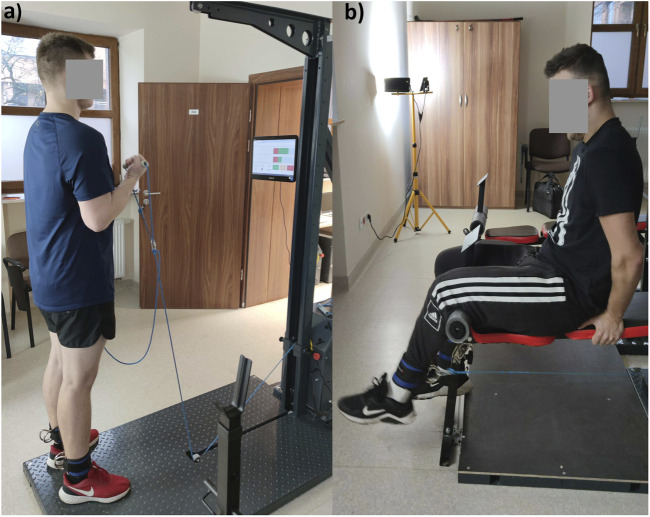
Participant positions during inertial training and testing; **(A)** elbow flexion, **(B)** knee extension.

Forty-two young males attended an initial recruitment meeting, and 30 agreed to participate in the study. Only volunteers who met the following inclusion criteria could participate in the study: in generally good health, at least 150 min of moderate physical activity per week for at least the last year, lack of professional training, and with a valid COVID-19 vaccination certificate. The exclusion criteria were: tendon or ligament injury in the previous 2 months and fractures in the previous 3 months. After applying these criteria, the study ultimately included 26 men (mean ± standard deviation: age 20.4 ± 1.18 years; body mass 81.0 ± 11.4 kg; height 184 ± 6.28 cm). The participants were physical education students, none of the participants was a competitive athlete. The participants were randomly allocated into two groups: inertial training group (IT, n = 13) or traditional gravity-dependent resistance training (TRT, n = 13) using the chit method, which is a simple method of generating random sequences. Both groups participated in 6 weeks of training, with IT performing inertial training and TRT performing traditional gravity-dependent resistance training. All participants were asked to maintain their standard diet and physical activity levels throughout the duration of the study. However, we did not control their lifestyle. All subjects provided written informed consent to participate in the study. Moreover, the participants showed in Figure 1, provided written informed consent for their images to be published. All procedures were approved by the local ethics committee (KB-UZ/34/2021), with approval based on the Declaration of Helsinki, and all methods were carried out in accordance with relevant guidelines and regulations. The participants and the public were not involved in the design, or conduct, or reporting, or dissemination plans of the research.

### 2.2 Estimation of training loads

To compare the effectiveness of the two types of training, we decided to use a novel method of determining loads in inertial training to make the training load in both training groups as similar as possible. Before training, participants learned exercise techniques in resistance and inertial conditions during two familiarization sessions. Then, 1RM was determined for unilateral elbow flexion and for unilateral leg extension (the exercises technique was the same as described in the training section). Next, the participants performed one set of unilateral flexion in the elbow joint (12 repetitions) and one set of unilateral extension in the knee joint with a load of 70% 1RM. During each set, the performance time was measured - the participants were encouraged to complete the set in the shortest time possible. Two days later, the training load for inertial training was determined using a Cyklotren inertial device (Inerion, Stanowice, Poland) according to Naczk et al. (2016b) recommendations. Each participant performed several sets of exercises with different loads. The load was increased or decreased so that the time to perform 12 repetitions (as fast as possible) was the same (with an accuracy of 0.5 s) as in the set time with 70% 1RM using traditional weights. If it was not possible to determine the load for inertial exercises using three sets with 5-minute breaks in between, the test was repeated the following day. The range of motion and body position of the participants during load determination were identical to those under resistance conditions.

### 2.3 Methodology limitation

We are aware that described above estimation of training loads and training protocols has some limitations. It should be clearly stated that in inertial training setting of training load, e.g., 70% of 1RM as in traditional weight training is not possible. In inertial training, the muscle load depends on both the speed of movement and the applied load. However, it should be noted that in inertial training using relatively light loads and high speed of movement, the muscle load (and fatigue) and post-training muscle strength increase may be equal or even greater compared to use larger loads and slower speed of movement (Naczk et al., 2014). We attempted to create a similar training protocol for both types of training. However, we recognize that these training protocols may have different effects on the body. In resistance training, failure fatigue appears at the end of the set, in inertial training after the first 3 repetitions - in each subsequent repetition the subject is able to accelerate the flywheel but develops less and less strength. However, in our opinion, the methodology of both trainings was as similar as possible. However, we have not found any attempt to directly compare the effectiveness of both types of training in the literature, so the methodology described above is original and may contain some flaws.

#### 2.3.1 Training

Both groups performed their training three times a week (Monday, Wednesday, and Friday) between 7:00 a.m. and 8:30 a.m. for 6 weeks. TRT performed traditional gravity-dependent resistance training using a weighted leg extension bench, while IT performed inertial training using the Cyklotren inertial device. Training was supervised by the same three researchers. Before each training session, standardized warm-ups were performed, either 5 min of submaximal cycling using upper and lower body ergometers with eight repetitions at 50% 1RM (TRT), or eight slow cycles with the Cyklotren device (IT). Each session trained two muscle groups: elbow flexors and knee extensors. Each exercise included three sets, with the right and left extremities being exercised separately. Twelve repetitions were performed in each set, and there was a 2-minute break between consecutive sets. A single training session lasted 20–25 min. Each exercise for the elbow flexors was performed in a standing position. In the starting position, the active arm was fully extended at the elbow joint. During inertial training of the elbow flexors, the participant held the handle connected to the rope, which was fully extended and tensed, with their hand in supination. To begin the exercise, the subject flexed his elbow. Each exercise for the knee extensors was performed in a seated position on a bench. In the starting position, the active leg was flexed at the knee joint to approximately 90°. To begin the exercise, TRT subjects straightened their knee moving the bench handle, while IT participant straightened their knee pulling the rope attached to the ankle. The range of motion was the same for both types of training: approximately 130° for the elbow flexors and approximately 80° for the knee extensors. Training loads were constance in both groups. We realize that the lack of load progression throughout the training was a limitation, however re-assessing training loads was troublesome. Even though the procedure of estimating 1RM is quick and easy, estimating the training load in IT would have been fatiguing (it sometimes took 2 days - see “Estimation of training loads”), which could have had a negative impact on the training process.

### 2.4 Measurements

Muscle strength was tested under different conditions before and after training. In addition, jumping ability, body composition, and limb circumference were evaluated. Measurements were taken on five separate days.

#### 2.4.1 1RM

1RM was determined for unilateral elbow flexion in a standing position using dumbbells and for unilateral leg extension in a seated position. The 1RM value was determined using traditional weights, according to National Strength and Conditioning Association (2008) guidelines. The participants performed a light warm-up set with 5–10 repetitions at 50% of estimated 1RM, followed by 2–3 heavier warm-up sets of 2–5 repetitions with loads increasing by 10%–20% at each set. Participants then began completing trials of 1 repetition with increasing loads (10%–20%) until they were no longer able to complete a single repetition. The highest load (kg) successfully lifted through the entire range of motion with the right arm with proper technique was denoted as the 1RM. Two min of rest were used between successive warm-up sets and 1RM trials.

#### 2.4.2 Measurement of maximal force under inertial conditions

The maximal force under inertial conditions (IFmax) was measured using the Cyklotren device (Naczk et al., 2015). The Cyklotren measurements exhibit very high reproducibility (intraclass correlation coefficient [ICC] consistency ≥0.969, ICC agreement ≥0.965). It should be noted that the participant’s position during IFmax measurements for both the elbow flexors and knee extensors was the same as during the 1RM test (Figure 1). Briefly, after warming up, each participant performed a 10 s maximal strength test for both elbow flexion and knee extension with the right and left extremities separately, with a 2-minute break between measurements. Estimated training loads were used during testing. The ranges of motion were approximately 130° for the elbow flexors and 80° for the knee extensors. The Cyklotren device displayed (on its screen) and recorded the force level for each repetition; the highest value of force (N) was used for future analysis.

#### 2.4.3 Maximal voluntary torque (MVT) measurement

The maximal torque derived from isometric muscle actions was determined using a specialized Biodex 4 Pro device (Shirley, NY, United States). Measurement methodology was similar to presented by Bezulska et al. (2018). Data collection was preceded by a familiarization session. Biomechanical measurements were collected in a seated position. During elbow flexor measurements, the hand of the active arm grasped the device handle, while the other hand remained on the abdomen. The shoulder and elbow joints of the active arm were set at 90 degrees of flexion. During knee extensor measurements, the ankle of the active leg was attached to the Biodex 4 Pro device moving shin pad. In the starting position, the thigh of the active leg was immobilized at 90° in relation to the trunk, and the knee was also positioned at 90°. To prevent any activity of other muscle groups that were not being tested, the participant’s trunk was stabilized using belts across the chest. Prior to the measurements, the participants were given verbal instructions regarding the experiment’s design. Each participant performed three maximal isometric contractions (for each tested muscle group for both the upper and lower extremities), each lasting 3 s and separated by 30 s breaks. The highest value among the three trials was adopted for further analysis.

#### 2.4.4 Jump tests

The vertical jump tests required each participant to perform three SJs (squat jumps), with a 30 s passive rest period between each effort, followed by three CMJs (countermovement jumps), with a 30 s passive rest period between each effort. Both the SJs and CMJs were performed using a TENDO JumpMat (Tendo Sports Machines, Trencin, Slovak Republic), jump mats can be successfully used to measure SJ and CMJ (Rogan et al., 2015). The highest of the three jump values (cm) was adopted for further analysis.

#### 2.4.5 Upper limb strength—Pull-ups

There are several different grip pull-ups, we used the type called chin-up, which strongly engages the arm flexor muscles (Johnson et al., 2009; Raizada and Bagchi, 2019). Participants grasped an overhead horizontal bar with their arms shoulder-width apart and forearms in the supinated position while hanging vertically (with feet just above the ground). The body was pulled upright in a linear path until the underside of the chin was level with or above the top surface of the horizontal bar. The participants were instructed to avoid all swinging, kicking, and twisting motions. Each participant had to perform as many repetitions as possible.

#### 2.4.6 Body composition

To evaluate the influence of training on body composition, a bioelectrical impedance device (Tanita MC-980 MA; Tanita Corporation, Tokyo, Japan) was used. The participants were asked to maintain a normal state of hydration prior to the measurements, and they were not allowed to exercise or eat for 12 h preceding the measurements. The measurements were made in the morning according to the manufacturer’s and Stahn et al. (2012) guidelines.

#### 2.4.7 Limb circumference

Upper arm circumference was measured at the thickest part of the arm in the tensed muscle (Hu et al., 2021). Thigh circumference was determined at the midpoint of the thigh of the loaded leg (Fukuoka et al., 2024). The same researcher took three measurements, with each made to the nearest 0.5 cm. The mean value of the three measurements was used for future calculations.

**FIGURE 2:**
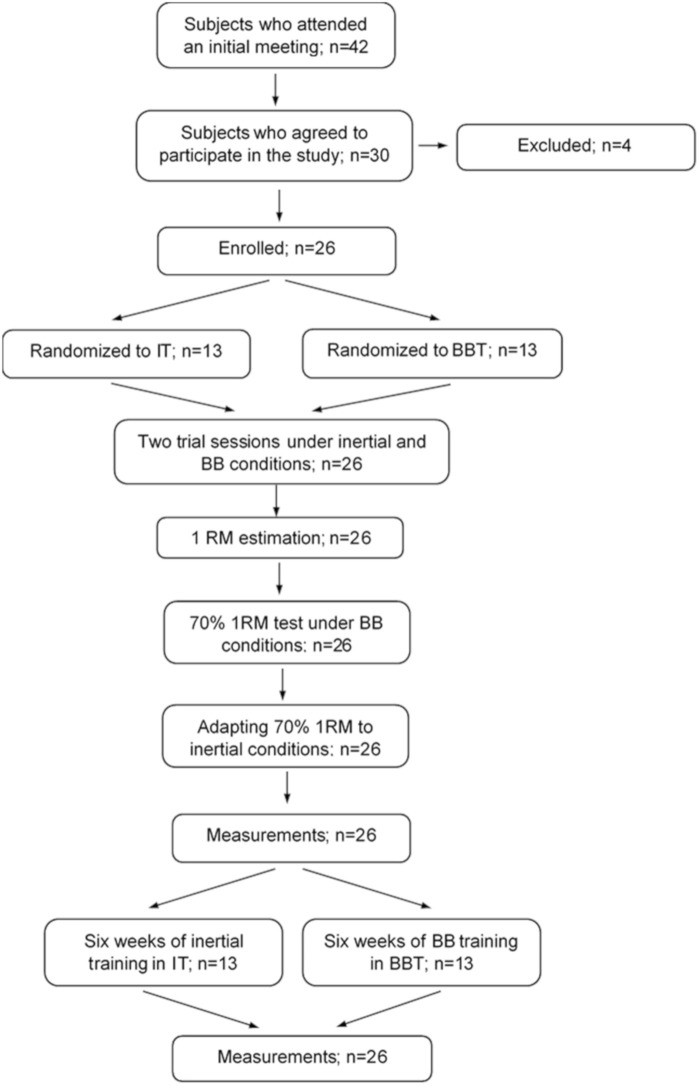
Flow diagram for study participants.

Visualization of a research sequence is presented in Figure 2.

### 2.5 Statistical analysis

The Shapiro–Wilk test was used to test if the data were normally distributed. Descriptive statistics, including means and standard deviations, were calculated. A two-way analysis of variance (ANOVA) with repeated measures was used to determine the effect of exercises. If differences were detected, the Scheffé *post hoc* procedure was used to determine where the differences occurred. The level of significance was set at P ≤ 0.05. The simple effect of training for each participant was defined as a relative increase in an analyzed variable after training compared with the value from before training. Lower and upper borders of 95% confidence intervals for relative increases were calculated. The effect size (ES) of the training was calculated using the independent two-sample *t*-test, and Cohen’s d was calculated. The scale presented by Cohen (1988) indicates that d < 0.41 represents a low ES, 0.41–0.70 represents a moderate ES, and values greater than 0.70 represent a high ES.

## 3 Results

None of the analyzed parameters differed significantly between the two tested groups at the beginning of the experiment. Muscle strength (1RM) increased significantly following training in both groups. It increased by 15%–22.6% in IT and 21.3%–27.3% in TRT However, the differences in relative strength increases between the two trained groups were not significant (Table 1).

**TABLE 1:** Mean and standard deviations for absolute values of 1RM and maximal force measured under inertial conditions.

Group/ muscle		1RM [kg]				IFmax [N]			
		EF		KE		EF		KE	
Limb		R	L	R	L	R	L	R	L
IT	Before	18.2 ± 1.71	17.9 ± 1.70	43.5 ± 5.33	43.5 ± 4.96	144 ± 19.6	144 ± 17.5	387 ± 66.9	385 ± 65.1
	95% CI	17.3–19.1	17.0–18.8	40.6–46.4	40.8–46.2	133–155	134–154	351–423	350–420
	After	20.9 ± 2.59*	20.8 ± 2.32*	53.5 ± 8.23*	53.5 ± 8.29*	185 ± 22.0*	185 ± 22.0*	460 ± 72.0*	488 ± 87.5*
	95% CI	19.5–22.3	19.5–22.1	49–58	49–58	173–197	173–197	421–499	440–536
	% change	15.0 ± 12.5	17.2 ± 14.7	22.3 ± 10.3	22.6 ± 9.93	29.8 ± 15.0	29.9 ± 16.0	19.7 ± 10.5#	27.1 ± 13.7#
IT vs. TRT	ES	0.55	0.28	0.32	0.31	0.38	0.30	1.51	1.59
TRT	Before	17.6 ± 2.43	17.6 ± 2.43	39.7 ± 6.53	39.4 ± 6.53	135 ± 22.8	135 ± 22.8	360 ± 59.1	367 ± 47.1
	95% CI	16.3–18.9	16.3–18.9	36.2–43.3	35.9–42.9	123–147	123–147	328–392	341–393
	After	21.3 ± 3.05*	21.2 ± 2.78*	49.8 ± 7.03*	49.4 ± 8.09*	164 ± 23.3*	166 ± 23.6*	343 ± 44.9	371 ± 65.1
	95% CI	19.6–23	19.7–22.7	46–53.6	45–53.8	151–177	153–179	319–367	336–406
	% change	21.8 ± 11.3	21.3 ± 13.9	27.0 ± 17.0	27.3 ± 17.9	23.7 ± 16.0	25.1 ± 14.6	−2.75 ± 17.4	1.73 ± 17.0

Notes: EF, elbow flexion; KE, knee extension; R, right limb; L, left limb; ES, effect size between groups, * *-* significant difference from baseline, # - significant difference between groups (P ≤ 0.05).

The IFmax of the elbow flexors increased significantly in both training groups, but the differences in relative force increases between IT and TRT were not significant. On the other hand, IFmax of the knee extensors increased significantly in IT only (by 19.7% and 27.1% in the right and left limbs, respectively), while changes in IFmax of the knee extensors in TRT were trivial (−2.75% and 1.73% in the right and left limbs, respectively). The relative increases noted in IT were significantly greater than in TRT, and the effect sizes expressed by Cohen’s d value were large (Table 1).

MVT increased significantly in all trained muscles in both IT and TRT (Table 2). The relative increases in elbow flexor torque were similar between the two groups, but the relative increases in knee extensor torque were slightly greater in IT compared to TRT; however, the differences were not statistically significant (ES = 0.41 and 0.51 for the right and left limbs, respectively, indicating a moderate effect).

**TABLE 2:** Mean and standard deviations for absolute values of MVT, CMJ, SJ, and maximal Pull-up test.

		MVT [nm]						
		EF		KE		CMJ [cm]	SJ [cm]	Pull-up [rep]
		R	L	R	L			
IT	Before	77.7 ± 10.1	72.4 ± 8.09	312 ± 57.9	277 ± 44.2	40.3 ± 5.60	32.7 ± 4.55	8.62 ± 5.06
	95% CI	72.2–83.2	68–76.8	281–344	253–301	37.3–43.3	30.2–35.2	5.87–11.4
	After	86.8 ± 12.4*	81.6 ± 12.0*	360 ± 58.1*	342 ± 60,3*	42.5 ± 3.72*	35.1 ± 3.80*	12.9 ± 5.86*
	95% CI	80.1–93.5	75.1–88.1	328–392	309–375	40.5–44.5	33–37.2	9.71–16.1
	% change	12.1 ± 12.1	12.7 ± 10.7	16.3 ± 10.1	23.6 ± 12.8	6.28 ± 8.10	7.95 ± 9.75	49.6 ± 36.2
IT vs. TRT	ES	0.27	0.02	0.41	0.51	0.17	0.32	0.41
TRT	Before	73.1 ± 10.6	68.5 ± 9.37	278 ± 54.2	270 ± 54.8	35.3 ± 6.25	29.6 ± 5.69	10.9 ± 5.28
	95% CI	67.3–78.9	63.4–73.6	249–308	240,300	31.9–38.7	26.5–32.7	8.03–13.8
	After	78.9 ± 10.7*	77.2 ± 12.3*	307 ± 53.5*	315 ± 67.1*	37.2 ± 7.86*	32.7 ± 6.34*	14.8 ± 6.92*
	95% CI	73.1–84.7	70.5–83.9	278–336	279 to 352	32.9–41.5	29.3–36.2	11–18.6
	% change	8.8 ± 11.7	12.9 ± 11.2	11.6 ± 12.2	17.2 ± 11.3	4,89 ± 7.40	11.0 ± 8.53	35.8 ± 27.6

Notes: EF, elbow flexion; KE, knee extension; R, right limb; L, left limb; ES, effect size between groups, * - significant difference from baseline, # - significant difference between groups (P ≤ 0.05).

The height of CMJ and SJ increased significantly in both training groups, and there were no significant differences in relative changes between groups (Table 2).

Elbow flexor muscle strength, evaluated by the pull-up test, increased significantly in both trained groups, but once again, the differences in relative changes between the two groups did not differ significantly (Table 3).

**TABLE 3:** Circumferences of the limbs.

	Circumferences of the limbs				
		Arms		Thighs	
		R	L	R	L
IT	Before	33.4 ± 2.99	33.2 ± 4.38	53.9 ± 3.60	53.9 ± 3.54
	95% CI	31.8–35	30.8–35.6	51.9–55.9	52–55.8
	After	34.4 ± 2.67*	34.4 ± 2.72*	55.1 ± 3.45*	55.0 ± 3.32*
	95% CI	32.9–35.9	32.9–35.9	53.2–57	53.2–56.8
	% change	3.23 ± 2.74	3.73 ± 2.92	2.13 ± 1.53#	2.04 ± 1.75#
IT vs. TRT	ES	0.09	0.10	1.38	1.15
TRT	Before	32.8 ± 2.77	32.6 ± 2.74	54.3 ± 4.04	54.2 ± 4.10
	95% CI	31.3–34.3	31.1–34.1	52.1–56.5	52–56.4
	After	33.9 ± 2.76*	33.9 ± 2.69*	54.5 ± 4.19	54.4 ± 4.15
	95% CI	32.4–35.4	32.4–35.4	52.2–56.8	52.1–56.7
	% change	3.44 ± 1.46	4.01 ± 2.27	0.34 ± 0.90	0.35 ± 0.99

Notes: R, right limb; L, left limb; ES, effect size between groups, * *-* significant difference from baseline, # - significant difference between groups (P ≤ 0.05).

Arm and thigh circumferences increased significantly following IT. However, in TRT, the circumference of the arms increased significantly, while changes in the circumference of the thighs were trivial (Table 3). The relative changes in the circumferences of the arms were similar between the two groups, but the relative changes in the circumferences of the thighs were significantly greater in IT than in TRT—ES = 2.13 and 2.04 for the right and left limbs, respectively.

Body composition did not change significantly in either training group.

## 4 Discussion

The results of this study indicate that changes in the elbow flexors following IT and TRT were similar. For every measurement condition (free weights, inertial conditions, isometric conditions, and pull-up test), strength levels increased significantly in both trained groups. There were no significant differences in relative strength increases between groups. In other words, the results showed that both types of training enhanced elbow flexor strength to a similar extent. No advantage to either training method was observed. To the best of our knowledge, no other studies have compared the effectiveness of inertial training vs. traditional resistance training in relation to elbow flexors. The relative increases in elbow flexor strength under inertial conditions in both groups were similar to those reported by Naczk et al. (2016b), who noted a 28.4% increase in elbow flexor strength after 5 weeks of inertial training.

Both inertial and traditional resistance training led to significant increases in knee extensor strength under free weight and isometric conditions, and the increases were similar in both groups. However, under inertial conditions, significant strength increases were noted in IT, while the TRT changes were trivial. Furthermore, ES showed an advantage for IT over TRT in strength enhancement under inertial conditions (ES = 1.51 and 1.59 for the right and left legs, respectively). It should be noted that considering the short training period (6 weeks), the increase in muscle strength (1RM and IFmax) in the IT (from 15% to 29.9%) was large. Increase in strength following inertial training may from both improvement in neuromuscular coordination and muscle hypertrophy. Significant increase of EMG amplitude was observed in Naczk et al. (2016a) in young males after 5 weeks of inertial training. In other study significant increase in EMG amplitude was noted just after 3 weeks of training performed by young males (Seynnes et al., 2007). Changes in EMG suggesting that significant neural adaptations occurred in response to the training stimulus and possibly indicating recruitment of higher threshold motor units. Moreover, Seynnes et al. (2007) can observed significantly increased quadriceps cross-sectional area just after 3 weeks of inertial training. Fast muscle mass increases may be due to a strong eccentric phase, which occurs during inertial training; eccentric contractions elicit greater muscle hypertrophy than concentric (Roig et al., 2009). However, the strength increases noted in IT under static conditions were not significantly greater than those in TRT, although ES indicated a slightly greater effect of inertial training compared to traditional resistance training (moderate ES according to Cohen (1988)). The strength improvements noted in IT (MVT = 16.3–23.6%) were greater than those observed by Onambélé et al. (2008), who reported an 8% increase in their inertial training group. Conversely, the improvements in TRT that we found in our study were similar to those reported by Onambélé et al. (2008), in their G-Weight group (traditional training). It should be mentioned that the cited authors trained elderly participants over a 12-week period. Our participants achieved greater improvements in knee extensor muscle strength than those observed by de Hoyo et al. (2015), who reported approximately 5% and 10% increases in maximal isometric voluntary contraction following traditional and inertial training, respectively. It should be noted, however, that body position, range of motion, and movement techniques in the two groups trained by de Hoyo et al. (2015) were different. The influence of traditional and inertial training on knee extensor muscle strength was also tested by Greenwood et al. (2007). They concluded that inertial training appeared to be as effective as standard resistance training for improving knee extensor muscle performance after knee injuries. However, in our opinion, the estimation of training loads for each training group was different.

Both training methods significantly improved vertical jump performance. Relative increases in CMJ and SJ in TRT and IT were similar. Improvement in jump tests may result both improvement in neuromuscular coordination and muscle hypertrophy. Moreover, may also result from increased muscle tendon stiffness (Onambélé et al., 2008), improvement of the stretch-shortening cycle (Bosco et al., 1981) and increase in the excitability threshold of the Golgi tendon organs (McNeely and Sandler, 2007). Similar improvement in CMJ and SJ in both groups noted in our study is contrary to results of de Hoyo et al. (2015), who stated that traditional training caused significantly greater improvement than horizontal inertial flywheel training. It can be caused by different movement technique and range of motion used during training in two groups of participants trained by de Hoyo et al. (2015) The traditional training group performed the half squat exercise on a smith machine when inertial training group performed a front step exercise using an inertial flywheel. Half squat training technique was similar to the CMJ technique. Therefore, the strength transfer to CMJ after half squat training was easier to achieve comparing to strength transfer from front step exercise. In our study movement technique and range of motion during training in both training groups were the same. Arm circumference increased significantly in both TRT and IT, while thigh circumference increased significantly in IT only (ES = 1.38 and 1.15 for the right and left thighs, respectively). This suggests that inertial training stimulated knee extensor hypertrophy more than traditional weight training. This is consistent with Norrbrand et al. (2008) and Norrbrand et al. (2010) conclusions; they stated that the greater mechanical stress that occurs during inertial training compared to traditional strength training may explain the robust muscle hypertrophy in response to inertial training. In general, both types of training were effective in relation to the tested parameters. Our results indicate that inertial training was as effective as traditional resistance training when the elbow flexors were considered. Our data are consistent with Vicens-Bordas et al. (2018a), who stated that inertial fiywheel resistance training and gravity-dependent resistance training improved muscle strength to a similar degree. However, in relation to knee extensor muscle strength, inertial training appeared to be slightly more effective than traditional resistance training. Among the six tested parameters (1RM, IFmax, MVT, CMJ, SJ, and limb circumference), two improved significantly more in IT compared to TRT (IFmax and thigh circumference). Moreover, the ES for MVT under isometric conditions was moderate, with a slight advantage for inertial training over traditional resistance training. This suggests that the effectiveness of the two types of training may vary depending on the different muscle groups being evaluated. We tested only two muscle groups, so future studies of other muscle groups are needed.

## 5 Limitations of the study

There are limitations to this study. First was described in methods section and concerning estimation of training loads and training protocols. Moreover, the training loads were not re-assessed throughout the training. Even though the procedure of estimating 1RM is quick and easy, estimating the training load in IT would have been fatiguing, which could have had a negative impact on the training process. Moreover, the study was conducted on a small group of participants. A small sample size limits the possibility of drawing strong conclusions. Second, we tested only two muscle groups (elbow flexors and knee extensors); it would be interesting to test the influence of the two training methods on other muscle groups. Another limitation of this research is its lack of data regarding longitudinal effects. We could not further evaluate the participants after the project was completed because of COVID-19 lockdowns. It would be interesting to evaluate how long the inertial and resistance training effects would be retained.

## 6 Conclusion

It can be stated that both types of training are highly effective in young physically active men, but the superiority of inertial training over traditional resistance training cannot be confirmed definitively, as Maroto-Izquierdo et al. (2017b) suggested in their review. Nevertheless, inertial training had a slight advantage over traditional resistance training when knee extensor muscle training was considered. It should be noted, the lack of definitive evidence that inertial training is superior to traditional resistance training does not mean that inertial training has no value as a training method. On the contrary, it is a very effective and useful training method for enhancing the locomotor system for sports (Davison et al., 2010; Maroto-Izquierdo et al., 2017a; Naczk et al., 2017) and rehabilitation (Fernandez-Gonzalo et al., 2016; Naczk et al., 2020; Naczk et al., 2022; Sarmiento et al., 2014). According to training principles for long-term training processes, varying training methods is often beneficial (Haff and Triplett, 2016). It is possible that inertial training can be a positive variation in one’s training regime when a plateau is reached. This is supported by Naczk et al. (2017), who concluded that even well-trained swimmers can significantly improve their muscle strength in a relatively short time by implementing a new variation to their training regime-inertial training. Nevertheless, the methodology of inertial training requires further development.

## Data Availability

The raw data supporting the conclusions of this article will be made available by the authors, without undue reservation.
